# Commentary: Considering intrauterine location in a model of fetal growth restriction after maternal titanium dioxide nanoparticle inhalation

**DOI:** 10.3389/ftox.2023.1293873

**Published:** 2023-12-04

**Authors:** John M. DeSesso, L. David Wise

**Affiliations:** ^1^ Exponent Inc, Alexandria, VA, United States; ^2^ Independent Teratologist, Philadelphia, PA, United States

**Keywords:** rat, fetal weight, normalized, adjusted, EFDT studies

## Introduction

We have read with interest the paper by D’Errico et al. (2021) and question the authors’ suggestion that fetal weights should be normalized based on their location within the uterus. Fetal weight effects are considered the most sensitive endpoint of embryo–fetal developmental toxicity (EFDT) studies (USEPA, 1991). Thus, it is important for a standard to be set for the calculation, presentation, and analysis of this endpoint.

To this end, it is well-established that the litter, rather than the fetus, is the experimental unit for analysis in EFDT studies (Jensh et al., 1970; Haseman and Hogan, 1975; USEPA, 1991; USEPA, 1998; OECD, 2018). Mean fetal weights are routinely calculated on a per litter basis wherein the mean fetal weight is calculated for each litter in a group, and then, these values are averaged to generate the overall group litter mean. Because male rat fetuses are generally heavier than females, mean fetal weights for each sex, calculated in the same general manner, are also typically reported. This method is considered the traditional dogma of fetal weight analyses.

An endpoint termed “normalized” or “adjusted” fetal weight is not described in regulatory guidance documents (e.g., USEPA, 1998; OECD, 2018). An inverse relationship between fetal body weight and litter size in polytocous species (although not absolute) has been recognized for many years (Romero et al., 1992) and should serve as the basis for developing methods that adjust fetal weights. The potential impact of uterine position on the distribution of nutrients and toxicants (and ultimately fetal health) has also been the subject of numerous investigations over the past 50 years (e.g., Barr et al., 1969; Barr et al., 1970; Ward et al., 1977; Norman and Bruce, 1979a; Norman and Bruce, 1979b; Padmanabhan and Singh, 1981). However, studies investigating the role of uterine position have produced conflicting results with regard to whether fetal weight is dependent or independent of horn size, horn side, and intrauterine position (Chahoud and Paumgartten, 2005).

## Comments on the normalized fetal weight formula

D’Errico et al. (2021) used a novel method to adjust fetal weights to account for the uterine horn side and the number of fetuses per horn, according to the following formula:

$$Normalized\;fetal\;weight=\frac{Fetal\;weight\;in\;g}{\left(GD20Maternal\;weight\;in\;g\right)÷\left(Number\;of\;fetuses\;in\;horn\right)}.$$

Several issues arise when considering this approach. First, they reported normalized fetal weights in g; however, inspection of the aforementioned formula discloses that the calculated value is unitless. Interestingly, historical control data for GD 20 “traditional” fetal weights average > 3.5 g (Charles River, 2023), while their unadjusted fetal weight (2.5 g) is much smaller and the normalized weights (0.05 g) are unrealistically diminutive. Second, neither the use of terminal maternal body weight in the equation to adjust the fetal weight is justified nor is any reference cited to support this approach. The authors suggest that “future studies should utilize maternal weight gain throughout pregnancy;” however, while these data are typically collected, the additional data will not improve their approach. Regardless, maternal body weight gain is driven largely by the total weights of the fetuses and, therefore, is not a basis to adjust fetal weights. Third, the sex of the fetuses was admittedly not taken into consideration, although this is an important factor in determining the fetal weight. Fourth, the mean adjusted fetal weight reports at four horn positions per litter were calculated from only four fetuses in some groups, which is too low to reach valid conclusions. Lastly, data from 22% of all right horns and 29% of all left horns were excluded because of a rule that excluded uterine horns with fewer than five fetuses (i.e., due to complications within the formula). The exclusion of a quarter of the data is remarkable and calls into question the validity of both this approach for analyzing fetal weights and the conclusions.

The authors state “However, upon further evaluation using our revised approach, we observed that under control conditions, feti [*sic*] implanted in the left uterine horn tended to be smaller in body weight at term compared with feti [*sic*] implanted in the right uterine horn.” We note that their statement is based on a low number of control litters (20), of which five right uterine horns and eight left uterine horns were excluded due to very few fetuses, and that their conclusion is contradictory to that reported by Barr et al. (1970).

## Conclusion

The approach taken by D’Errico et al. is indeed a challenge to accept as an alternative to the common dogma of litter data analysis. Given the numerous points raised above, we do not agree that this method in its present form allows for more refined conclusions to be drawn from fetal weight data. The substantial amount of the data that must be excluded to draw those conclusions from guideline-compliant EFDT studies would likely adversely impact human hazard identification.
